# The Complete Maternally and Paternally Inherited Mitochondrial Genomes of a Freshwater Mussel *Potamilus alatus* (Bivalvia: Unionidae)

**DOI:** 10.1371/journal.pone.0169749

**Published:** 2017-01-09

**Authors:** Hai B. Wen, Zhe M. Cao, Dan Hua, Pao Xu, Xue Y. Ma, Wu Jin, Xin H. Yuan, Ruo B. Gu

**Affiliations:** 1 Wuxi Fishery College, Nanjing Agriculture University, Jiangsu, China; 2 Key Laboratory of Genetic Breeding and Aquaculture Biology of Freshwater Fishes—Ministry of Agriculture, Freshwater Fisheries Research Center, Chinese Academy of Fishery Sciences, Jiangsu, China; 3 Sino-US Cooperative Laboratory for Germplasm Conservation and Utilization of Freshwater Mollusks, Freshwater Fisheries Research Center, Chinese Academy of Fishery Sciences, Jiangsu, China; University of Cambridge, UNITED KINGDOM

## Abstract

Doubly uniparental inheritance (DUI) of mitochondrial DNA, found only in some bivalve families and characterized by the existence of gender-associated mtDNA lineages that are inherited through males (M-type) or females (F-type), is one of the very few exceptions to the general rule of strict maternal mtDNA inheritance in animals. M-type sequences are often undetected and hence still underrepresented in the GenBank, which hinders the progress of the understanding of the DUI phenomenon. We have sequenced and analyzed the complete M and F mitogenomes of a freshwater mussel, *Potamilus alatus*. The M-type was 493 bp longer (M = 16 560, F = 16 067 bp). Gene contents, order and the distribution of genes between L and H strands were typical for unionid mussels. Candidates for the two ORFan genes (*forf* and *morf*) were found in respective mitogenomes. Both mitogenomes had a very similar A+T bias: F = 61% and M = 62.2%. The M mitogenome-specific *cox2* extension (144 bp) is much shorter than in other sequenced unionid mitogenomes (531–576 bp), which might be characteristic for the *Potamilus* genus. The overall topology of the phylogenetic tree is in very good agreement with the currently accepted phylogenetic relationships within the Unionidae: both studied sequences were placed within the Ambleminae subfamily clusters in the corresponding M and F clades.

## Introduction

Doubly uniparental inheritance (DUI), widespread through the class Bivalvia, is one of the most striking exceptions to the general rule of strict maternal inheritance of mitochondrial DNA (mtDNA) in animals [1]. DUI is an ancient system, likely around 500 million years old [2], characterized by the existence of gender-associated mtDNA lineages that are inherited through males (M type) or females (F type). Females are homoplasmic: they have only the F type mtDNA, inherited from their mothers, which they pass on to both female and male offspring. Males are heteroplasmic: they possess both M and F type; the former is received from the father and transmitted only to the male offspring, whereas the F type is inherited from the mother and not transmitted to the next generation. The M type is found predominantly in male gonads, while the F type is more abundant in somatic tissues. The two mitochondrial genomes are of similar size, comparable to mammalian mtDNA. They evolve faster than mtDNA in most other animals, and M type evolves faster than the F type (reviewed in [3–6]). Whereas marine bivalves under the DUI undergo occasional masculinisation events (a loss of the M mitogenome and its replacement with the F mitogenome) [7], the two mitogenomes in Unionidae family have been evolving independently for about 200 million years. This is believed to be a consequence of the M-specific extension of the *cox2* gene, which prevents masculinisation events [8–10].

Compared to marine bivalves (particularly Mytilidae), freshwater species are less thoroughly studied with respect to DUI [4]. Bivalves of the order Unionoida are the largest group of freshwater bivalves, comprising six families and over 800 species distributed across six continents [11]. DUI has been described in over 60 of these species [4]. With 442 Unionidae species currently included in the IUCN Red List of Threatened Species [12], freshwater mussels are probably the most endangered group of freshwater animals today [13]. As the importance of their role in the maintenance of healthy freshwater ecosystems could not be overstated [14], there exists a pressing need for a better understanding of the ecology and biology (including genetics) of this understudied group of animals.

DUI is not only an excellent model system for studying the evolutionary history and phylogeographic structure of freshwater mussels, but also for studying the evolution of mtDNA genomes, and particularly the evolutionary forces that maintain the strictly maternal mtDNA inheritance in most other animals [3,15,16]. More than 50 unionid mitogenomes have been sequenced to date, e.g. [3,17–20], but none belonging to *Potamilus* genus. Furthermore, as researchers often use only somatic tissues as the source of DNA, and thus obtain only the F type mtDNA, this results in the underdetection of M-type mitogenomes and DUI [4]. Consequently, M type sequences are underrepresented in the GenBank, with only ten currently available. As this hinders the progress of the understanding of the DUI phenomenon, we have identified, sequenced, characterized, compared and phylogenetically analysed both F and M mitogenomes of a freshwater mussel, the pink heelsplitter *Potamilus alatus* (Unionidae: Ambleminae). This aquatic bivalve mollusk, native to North America, was introduced to China in 2012 as a viable candidate for the production of atropurpureus freshwater pearls [21,22], so apart from contributing to a better understanding of the DUI, the publication of the complete mtDNA sequences shall be useful for the development of the breeding technology for this species.

## Materials and Methods

### Ethics statement

All necessary permits were obtained from the Taihu Lake Fishery Administration, Jiangsu Province. The handling of mussels was conducted in accordance with the guidelines for the care and use of animals for scientific purposes set by the Institutional Animal Care and Use Committee of the Nanjing Agricultural University, Jiangsu, China.

### Samples and DNA extraction

*P*. *alatus* samples, originating from the stock introduced to China in 2012 from the Virginia Institute of Technology [21], were obtained at the Nanquan experimental base of the Freshwater Fisheries Research Center, Chinese Academy of Fishery Sciences (120°16’ E, 31°25’ N). Determining the presence and characteristics of DUI (mainly the M haplotype) in freshwater bivalves requires the isolation of DNA from male gonads and somatic tissues of both sexes [4]. Hence, specimens were first sexed based on the appearance of the gill marsupium during the reproduction period, when gravid females fill and expand the outer gills with glochidia, whereas in males they remain flat [23]. Afterwards, gonads of the preliminary sexed specimens were dissected and examined under a microscope to further verify the sex of the specimens. Five tissues (foot, gonads, mantle, gills and adductor muscle) of three females and three males were selected for the DNA extraction. After the presence and the ratio of the two types of mtDNA in tissues were determined by a preliminary amplification and sequencing of the *cox1* gene [16], the complete mitogenomes were amplified from the adductor muscle sample of a selected female specimen and from the gonads of a selected male specimen. Both of these specimens are stored at the Freshwater Fisheries Research Center, Department of Aquaculture, Wuxi, under accession numbers N120F (F) and N466M (M). DNA was extracted using the Aidlab DNA extraction kit (Aidlab Biotechnologies, Beijing).

### PCR amplification and mitogenome sequencing

Degenerate primers (Table 1) were designed to match the generally conserved regions of mtDNA genes and used to amplify and sequence short fragments of *12s*, *cox1*, *cox3*, *nad4*, *cytb*, *nad1* and *nad2* genes. These sequences were then used to design the specific primers for the amplification and sequencing of the remaining mitogenomic sequences in several PCR steps. Primers were designed to amplify products with overlaps of about 100 bp. Sequences were assembled in a stepwise manner, ensuring that the overlaps are identical. All obtained fragments were BLASTed [24] to confirm that the amplicon is the actual target sequence. Reaction volume of 50 μL contained 5 U/μL of TaKaRa LA Taq polymerase (TaKaRa, Japan), 10×LATaq Buffer II (Mg2+Plus), 2.5 μM of dNTP mixture, 0.2–1.0 μM of each primer, 60 ng of DNA template, and PCR-grade H2O. PCR conditions were optimized for each reaction, with the annealing temperature adjusted to suit the specific primer pair, extension time set to 1 min per Kb of the expected product size, and cycles (35 on average) adjusted depending on the amplification efficiency of the primers. PCR products were sequenced directly, using Sanger method, on an ABI 3730 automatic sequencer. When that was not possible, the products were cloned into a pMD18-T vector (TaKaRa, Japan) and then sequenced. DNAstar v7.1 (Dnastar Inc., USA) package was used to assemble the sequences and locate the putative ORFs for protein-coding genes. Then we used BLAST and Blastx to compare the putative ORFs with the published nucleotide and amino acid sequences of related species, and manually determine the actual initiation and termination codon positions. Annotation of tRNAs was performed using tRNAscan [25] and ARWEN [26] tools and the results checked manually. GenBank accession numbers for the mitogenomes are KU559011 (F) and KU559010 (M). Genomes were visualized using CLC Sequence Viewer 7.7.1 (QIAGEN Bioinformatics).

**Table 1:** Primers, degenerate and specific, used for the PCR amplification of *Potamilus alatus* male (M) and female (F) type mitochondrial genomes.

Fragment No.	Gene or region	Primer name	Sequence (5’-3’)	Length (bp)
F1	*cox3*	HBCOX3F	ACRTCNACAAARTGYCARTAYCA	342
		HBCOX3R	GCTGTDTTDYTDGGBTCHGGDGT	
M1	*cox1*	HBCOX1F	CTAARCAHTTYAAYCCDAGYAGG	938
		HBCOX1R	ACYAATCRTAARGAYATTDGGAC	
F2/M2	*nad2*	HBND2F	AAYTGRYTAAYHACHTGRATWGG	308
		HBND2R	GGDGCRATYTTTTGYCADGT	
F3/M3	*12S*	HB12SF	TGGTGCCAGCAKTCGCGGT	600
		HB12SR	ACYCCYACTDTGTTACGACTT	
F4/M4	*cytb*	HBCYTBF	GGBTAYGTNCTHCCNTGRGGNCA	434
		HBCYTBR	AARTAYCAYTCDGGYTGAATRTG	
F5	*nad5*	HBND5F	AAHAADGCYTTAAANANHGCRTG	314
		HBND5R	GCDGCNATRGCHGCYCCWACNCC	
M5	*nad1*	HBND1F	GCNCAAARAAYTTDATACGARGT	407
		HBND1R	ATDAGNARATCRTAGBGRTAACRCGG	
M6/F6	*nad4*	HBND4F	GGNGCYTCNRCATGVGCYTTNGG	251
		HBND4R	GGNTGRGGNTAYCADCCDGARCG	
F7	*cox3-nad2*	2HBF1	TACACCTAAGAACCTCACC	4741
		2HBR1	TGTTGGGTGAAGAGAGGAG	
F8	*cox3-nad2*	2HBF1	TACACCTAAGAACCTCACC	4741
		2HBR1	TGTTGGGTGAAGAGAGGAG	
F9	*nad2-12S*	2HBF2	CAGACCCCAACCTATGGTC	545
		2HBR2	TCCCATGGGACAGCTTTCC	
F10	*12S-cytb*	2HBF3	CTAAAGCGAAATCTAGTAC	1573
		2HBR3.5	AGAATCCTCCTCACAGCC	
F11	*cytb-nad5*	2HBF3.1	ACACTAACCTCCATTTGCC	1582
		HBR3	AACTCTTAGGCAGCTTGGTC	
F12	*nad5-nad4*	HBF4	TCACAAAATCTCTGGACTCTC	2226
		2HBR5	TGGTGATGAGGAAACGTAAG	
F12	*nad4-cox3*	2HBF6	ACAAAACACTACACTAATCG	2097
		2HBR6	TTAGATATCTTGTGTTGGTG	
M7	*cox3*	HBCOX3F	ACRTCNACAAARTGYCARTAYCA	342
		HBCOX3R	GCTGTDTTDYTDGGBTCHGGDGT	
M8	*cox3-cox1*	1HBF8	CTCAAAAACTTGTAATACGG	*1327*
		HBR7	CTTAGAATTGGGGTGATAGG	
M9	*cox1-nd2*	HBF6	AACTGGTCATCACACATCAC	*1543*
		1HBR6-1	AGTGTATGTGGGTTTGTG	
M10	*nad2-12S*	1HBF1	ATCCAGACCACTAACAAG	*1361*
		1HBR1	GTTTGGGTGTAAACTTAAGG	
M11	*12S-cytb*	1HBF2-0	AAGTAAGACCACAACACT	*2258*
		1HBR2	AGAAGTCTTGTTCCAGAC	
M12	*cytb-nad1*	2HBF4	ACGAAACATACACATGGC	*3975*
		13HB-B	GGTTAGAATTATAGGAATTTCG	
M13	*nad1-nad4*	13HBF4	GATTCAACGTAGAATACTCAAGAG	*1686*
		1HBR4	TGGAGTTCTTTGGAGTGAG	
M14	*nad4-cox3*	1HBF5	ATAGCTTCATAACACAAACC	*2650*
		1HBR7	TGTTAGTGTGTTTGTGACG	

### Phylogenetic analyses

To provide additional evidence that our samples were correctly identified, and that sequencing and annotation were conducted with high precision and reliability, we have selected a subset of unionid mitogenomes available from the GenBank and conducted phylogenetic analyses. To maximise the phylogenetic resolution for the two studied sequences, we have included all of the available sequences belonging to the Ambleminae subfamily; two M mitogenomes: *Venustaconcha ellipsiformis* and *Quadrula quadrula* [3]; and five F mitogenomes: *Toxolasma parvus* [27], *Leptodea leptodon* [28], *Lampsilis ornata* [17], *Venustaconcha ellipsiformis* and *Quadrula quadrula* [3]. Additional twelve unionid sequences, two Margaritiferidae (Bivalvia: Unionoida), and a *Mytilus edulis* (Bivalvia: Mytilioda) sequence as outgroup were also retrieved from the GenBank. Analyses were performed on concatenated twelve mitochondrial protein-coding genes and two rRNA genes. *Atp8* was not used in the analysis because it is not present in all unionid mitogenomes [3]. MitoTool (https://github.com/dongzhang0725/MitoTool) package was used to retrieve the sequences from the GenBank. Each sequence was aligned separately (in batches) by MAFFT [29] integrated into BioSuite program (https://github.com/dongzhang0725/BioSuite): rRNAs were aligned directly, whereas PCGs were translated into amino acid sequences (by MitoTool), aligned (MAFFT), and then back-translated into the corresponding codon-based DNA alignments using pal2nal.pl [30]. Alignments were visually inspected and ambiguously aligned parts manually deleted. Finally, BioSuite was used to concatenate the alignments (S1 File) and produce input files for the two programs used to conduct the phylogenetic analysis: maximum-likelihood (with 1000 bootstrap replications) using raxmlGUI [31,32], and Bayesian inference using MrBayes 3.2.6 [33], with 4×10^6^ generations. GTR+G+I evolution model, selected using Modelgenerator [34], was used in both analyses.

## Results and Discussion

### Genome size and organization

**Fig 1:**
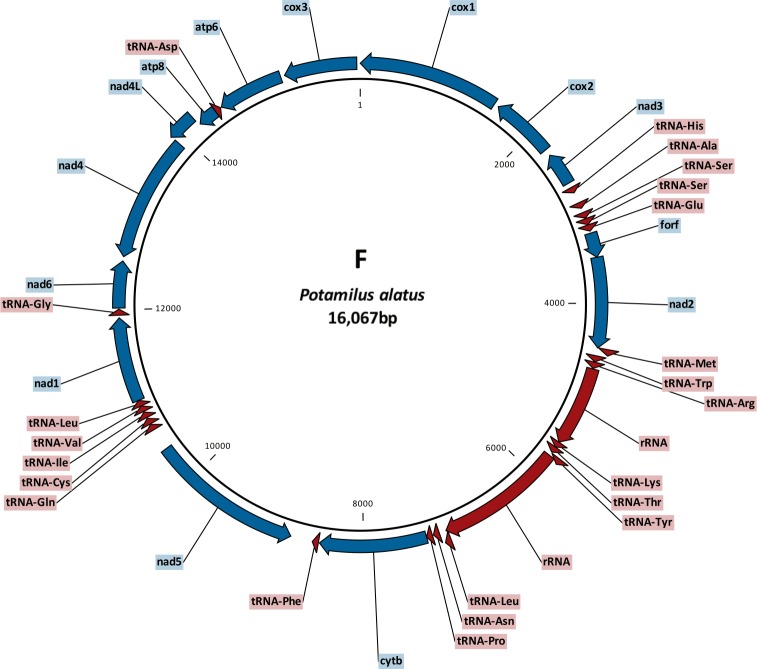
Gene map of the F type mitochondrial genome of *Potamilus alatus*.

**Fig 2:**
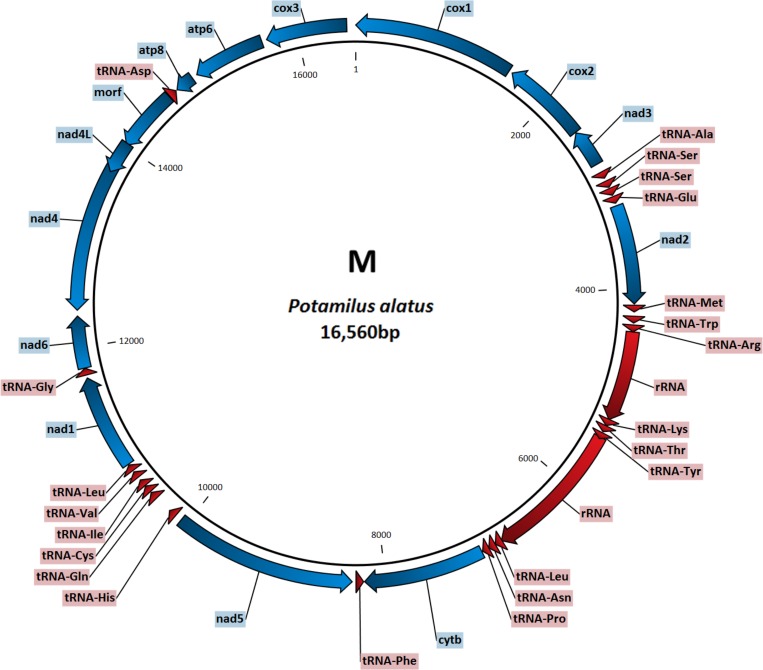
Gene map of the M type mitochondrial genome of *Potamilus alatus*.

In unionid bivalves, the M mitogenome is usually about 550 bp longer than the F type, mostly as a result of the M-specific extension of *cox2* gene [4,8]. In *P*. *alatus*, the M genome (16 560 bp) is also longer than the F genome (16 067 bp), but the difference is only 493 bp. The F genome is somewhat larger than the usual unionid F mitogenomes, whereas the M genome is shorter than most other unionid counterparts [18,20,35–39]. Both genomes contained all 37 genes commonly found in animal mtDNAs [40]: 13 protein-coding genes, 22 tRNAs (including double serine and leucine tRNAs) and two (12S and 16S) rRNAs (Figs 1 and 2, S1 Table and S2 Table). Control region was not found, but candidates for two ORFan [3,41] genes (*forf* and *morf*) were found in respective genomes. The gene order in both genomes, as well as the distribution of genes between L and H strands, was typical for unionid mussels [3]. Non-coding regions ranged from 2 to 233 bp in length in the F mitogenome (26 in total) and from 1 to 170 bp in the M mitogenome (31 in total). These numbers are within the standard range (22–33) of unassigned regions usually found in unionoid mitogenomes [3,17,36,37,42]. Two gene overlaps were found in the F genome: *nad2* / *tRNA*^*Met*^ (1 bp) and *nad4* / *nad4L* (8 bp). There is a possibility that *nad2* uses the unfinished T—top codon instead of TAA, in which case it does not overlap with the *tRNA*^*Met*^. Overlap between *nad4L* and *nad4* is common in unionoid mitogenomes [3,4]. Two gene overlaps were found in the M genome as well: 3 bp between 12S rRNA and tRNA^Lys^ and 17 bp between *nad4L* and *morf*. Gene overlaps in mitogenomes usually involve tRNA genes, because their sequences are under lesser evolutionary constraints [43]. However, the large (17 bp) overlap between *nad4L* and *morf* indicates the possibility of an annotation mistake. We have compared the translated *morf* amino acid product with the remaining four *morf* sequences available in the GenBank, and the results indicate the existence of four extra bases at the end of the *P*. *alatus morf* polypeptide. However, this would require GGA to be used as the stop codon, which is not likely. It is also possible that such a large overlap was made possible by the low functional constraints on *morf* evolution, as evidenced by very low level of sequence similarity between the available orthologs [3]. This problem further highlights the need for a larger number of fully annotated unionid M type genomes to be available in public databases. Four different start codons were found in the F genome: AUG (6 genes), AUU (5), GUG (2), AUA (1); and two stop codons: UAG (8) and UAA (6). Three start codons were found in the M genome: AUG (9 genes), AUU (3), AUA (2); and two (complete) stop codons: UAA (8), UAG (5). We did not count U—stop codon separately, as we presumed that it would be completed (UAA) by the addition of 3' A residues to the mRNA [17,44–46]. All these codons are typical for the invertebrate mitochondrial DNA [40].

### Base composition

A+T bias has been observed in many unionid mitogenomes [4], and the two *P*. *alatus* genomes are no exception: F = 61% (38% A, 23% T, 11% G, 28% C) and M = 62.2% (39.2% A, 23.0% T, 10.6% G, 27.2% C). Almost identical A and G ratios were observed in the F mitogenome of *U*. *pictorum* [35]. There was very little variation in base composition among different functional parts of the genome: *cox2* had the lowest A+T bias in both genomes (56.2% in F, 58.9% in M), whereas *nad3* (63.0%) and *nad6* (63.9%) had the highest bias in F and M genomes, respectively.

### ORFan genes

All bivalve mollusks under DUI appear to possess lineage-specific mitochondrial ORFs (open reading frames). Their origin in bivalves may be extremely ancient, it is believed that *forf* has been maintained for at least 200 million years in unionids [1,4,41]. Even though both genes appear to be rapidly evolving [3], their long-term conservation in bivalves, as well as their transcriptional levels, suggest that they are functional: they could be directly involved in sex determination, reproduction, embryo development, spermatogenesis, or just the maintenance of sperm mitochondria during the embryo development [1,5,27]. We have found a 264 bp-long candidate for the *forf* gene between *nad2* and *nad3* genes in the studied F mitogenome, and a 612 bp-long candidate for the *morf* gene between *nad4l* and *tRNA*^*Asp*^ in the M genome. This is standard for ORFan genes: their position generally differs between F and M genomes and their length varies from 66 to 92 codons (198 to 276 bp) in F genomes and from 101 to 234 codons (303 to 702 bp) in M genomes (reviewed in [5,47]).

### Gene sizes

Assuming that there were no major annotation oversights, a somewhat larger number of longer genes was found in the M genome, but the differences in size for most (26) of the genes were in a single-digit range. Hence, the extra 493 bases in the male genome can be attributed mostly to the differences in sizes between *cox1* (28 bp), *cox2* (144 bp), *nad5* (84 bp), and *morf/forf* (348 bp) genes. Only one gene, *atp8*, was much larger in the F genome: 27 bp.

### *cox2* extension

Unionid M genomes are characterised by the presence of a unique 3’-end coding extension of the cytochrome c oxidase subunit II (*cox2*) gene [8]. It is functional, rapidly evolving and its absence from the F genome suggests that it plays an important role in the mechanism of DUI [3,47]. It usually spans 531 to 576 bases [4,9,19], but in *P*. *alatus*, the extension is much shorter, only 144 bases: F *cox2* is 681 bp-long, whereas the M *cox2* is 825 bp-long. As an identical (144 bp) extension was also found in the closely related *Potamilus purpuratus* [9], this significantly shortened extension might be characteristic for this genus.

### Similarity and phylogeny

**Fig 3:**
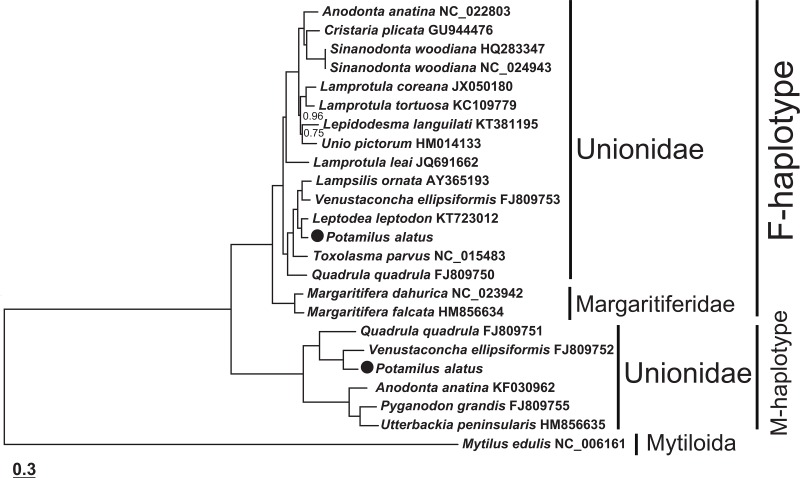
Phylogenetic positions of the two studied *P*. *alatus* mitogenomes.

Unionoid bivalves are characterized by extreme intraspecific sequence divergences between gender-associated mtDNAs [10]. Both synonymous and non-synonymous substitutions accumulate faster in the M genome. As expression studies indicate that the M genome is active only at spermatogenesis, these observations suggest that the M genome is under a more relaxed selective constraint than the F genome [3,47,48]. Whereas several masculinisation events are believed to have occurred during the evolutionary history of Mytillidae, the two mitogenomes have been evolving independently in Unionidae for at least 200 million years [4,7–9,47]. As the divergence between male and female genomes starts *de novo* with each masculinisation event, the genetic divergence between F and M genomes in the marine genus *Mytilus* is only 21–26% [49,50], whereas in Unionidae it is usually in the range of 40 to 50% [10,19]. The genetic divergence between the two *P*. *alatus* genomes (40%) is at the lower end of this range. As a result of the high diversity and the absence of recent masculinisation of mtDNA in freshwater bivalves, separate F and M clades are formed in phylogenetic analyses and can be independently used for phylogeny reconstruction [9,47]. The two approaches (Maximum Likelihood and Bayesian inference) used in this research to estimate the phylogenetic position of *P*. *alatus* produced identical dendrogram topologies (consensus tree is shown in Fig 3) with very high statistical support. The overall topology of the phylogenetic tree is in very good agreement with the results of the recent review of the phylogenetic relationships within the Unionidae based on *cox1* and *28S* genes [51]: both studied sequences were placed within the Ambleminae subfamily clusters in the corresponding M and F clades. Furthermore, the topology of both clades corroborates the close relationships of *Potamilus* with other Lampsilini tribe genera included in the analysis: *Leptodea*, *Lampsilis*, *Venustaconcha* and *Toxolasma* [51,52].

Phylogenetic dendrogram showing the evolutionary relationships among 24 almost complete bivalve mtDNA sequences: 21 Unionidae (including *P*. *alatus*), two Margaritiferidae and *Mytilus edulis* (outgroup). Scale bar corresponds to the estimated number of substitutions per site. Statistical support values were all 100 (not shown), except where noted otherwise: Bayesian posterior probability values (Bayesian inference analysis) are bolded and larger, bootstrap support values (Maximum Likelihood) are italicized and smaller. *Potamilus alatus* sequences are highlighted by a black dot. GenBank accession numbers are indicated in the figure.

## Conclusions

Both M and F *Potamilus alatus* mitogenomes are relatively similar to other described unionid mitogenomes, and phylogenomic analysis results are in good agreement with the phylogenetic relationships inferred using other molecular markers. The publication of this sequence shall be valuable for the future studies of the DUI phenomenon in bivalves. Apart from focusing on further identification and sequencing of (still) underrepresented M genomes in freshwater mussels, future studies should also further explore the phenomenon of the shortened *cox2* extension in the M genome of the *Potamilus* genus.

## Supporting Information

S1 File The alignment of 24 mtDNA sequences used for phylogenetic analyses. Sequences include concatenated twelve mitochondrial protein-coding genes (*Atp8* was not used in the analysis) and two rRNA genes.
(FAS)

S1 Table Structural features of the F-type *Potamilus alatus* mitochondrial genome. Gene lengths are in bp, St = strand, and NCR = non-coding region, where a negative value indicates an overlap between two genes.
(DOCX)

S2 Table Structural features of the M-type *Potamilus alatus* mitochondrial genome. Gene lengths are in bp, St = strand, and NCR = non-coding region, where a negative value indicates an overlap between two genes.
(DOCX)
