# Supplementary material for: The Complete Maternally and Paternally Inherited Mitochondrial Genomes of a Freshwater Mussel Potamilus alatus (Bivalvia: Unionidae)
Source: PLoS One. 2017 Jan 9;12(1):e0169749. doi: 10.1371/journal.pone.0169749 (PMC5222514; doi:10.1371/journal.pone.0169749)
Supplement: S1 Table — Gene lengths are in bp, St = strand, and NCR = non-coding region, where a negative value indicates an overlap between two genes. (DOCX) [file pone.0169749.s002.docx]

| **Gene** | **Position** | | **Size** | **IGN** | **Codon** | | **Anti-**  **Codon** | **St** |
| --- | --- | --- | --- | --- | --- | --- | --- | --- |
|  | From | To |  |  | Start | Stop |  |  |
| *cox1* | 1 | 1509 | 1509 |  | ATT | TAG |  | H |
| *cox2* | 1558 | 2238 | 681 | 48 | ATG | TAG |  | H |
| *nad3* | 2311 | 2667 | 357 | 72 | ATG | TAG |  | H |
| tRNA-His | 2721 | 2784 | 64 | 53 |  |  | GTG | H |
| tRNA-Ala | 2902 | 2965 | 64 | 117 |  |  | TGC | L |
| tRNA-Ser2 | 3014 | 3078 | 65 | 48 |  |  | TGA | L |
| tRNA-Ser1 | 3085 | 3152 | 68 | 6 |  |  | TCT | L |
| tRNA-Glu | 3156 | 3224 | 69 | 3 |  |  | TTC | L |
| *forf* | 3247 | 3510 | 264 | 22 | ATT | TAA |  | L |
| *nad2* | 3511 | 4476 | 966 |  | ATG | TAA |  | L |
| tRNA-Met | 4476 | 4540 | 65 | -1 |  |  | CAT | L |
| tRNA-Trp | 4562 | 4626 | 65 | 21 |  |  | TCA | L |
| tRNA-Arg | 4631 | 4698 | 68 | 4 |  |  | TCG | L |
| 12S rRNA | 4699 | 5563 | 865 |  |  |  |  | L |
| tRNA-Lys | 5564 | 5626 | 63 |  |  |  | TTT | L |
| tRNA-Thr | 5629 | 5692 | 64 | 2 |  |  | TGT | L |
| tRNA-Tyr | 5701 | 5763 | 63 | 8 |  |  | GTA | L |
| 16S rRNA | 5764 | 7088 | 1325 |  |  |  |  | L |
| tRNA-Leu1 | 7089 | 7153 | 65 |  |  |  | TAG | L |
| tRNA-Asn | 7180 | 7247 | 68 | 26 |  |  | GTT | L |
| tRNA-Pro | 7258 | 7322 | 65 | 10 |  |  | TGG | L |
| *cytb* | 7323 | 8469 | 1147 |  | ATT | T-- |  | L |
| tRNA-Phe | 8480 | 8547 | 68 | 11 |  |  | GAAA | L |
| *nad5* | 8781 | 10451 | 1671 | 233 | ATG | TAA |  | H |
| tRNA-Gln | 10661 | 10726 | 66 | 209 |  |  | TTG | L |
| tRNA-Cys | 10733 | 10799 | 67 | 6 |  |  | GCA | L |
| tRNA-Ile | 10810 | 10874 | 65 | 10 |  |  | GAT | L |
| tRNA-Val | 10883 | 10946 | 64 | 8 |  |  | TAC | L |
| tRNA-Leu2 | 10947 | 11010 | 64 |  |  |  | TAA | L |
| *nad1* | 11011 | 11913 | 903 |  | ATT | TAA |  | L |
| tRNA-Gly | 11942 | 12005 | 64 | 28 |  |  | TCC | L |
| *nad6* | 12018 | 12518 | 501 | 12 | ATA | TAA |  | L |
| *nad4* | 12565 | 13911 | 1347 | 46 | ATT | TAG |  | H |
| *nad4L* | 13904 | 14200 | 297 | -8 | GTG | TAG |  | H |
| *atp8* | 14216 | 14425 | 210 | 15 | GTG | TAA |  | H |
| tRNA-Asp | 14426 | 14491 | 66 |  |  |  | GTC | H |
| *atp6* | 14502 | 15209 | 708 | 10 | ATG | TAG |  | H |
| *cox3* | 15253 | 16032 | 780 | 43 | ATG | TAG |  | H |
