# Supplementary material for: The Complete Maternally and Paternally Inherited Mitochondrial Genomes of a Freshwater Mussel Potamilus alatus (Bivalvia: Unionidae)
Source: PLoS One. 2017 Jan 9;12(1):e0169749. doi: 10.1371/journal.pone.0169749 (PMC5222514; doi:10.1371/journal.pone.0169749)
Supplement: S2 Table — Gene lengths are in bp, St = strand, and NCR = non-coding region, where a negative value indicates an overlap between two genes. (DOCX) [file pone.0169749.s003.docx]

| **Gene** | **Position** | | **Size** | **IGN** | **Codon** | | **Anti-**  **Codon** | **St** |
| --- | --- | --- | --- | --- | --- | --- | --- | --- |
|  | From | To |  |  | Start | Stop |  |  |
| *cox1* | 1 | 1537 | 1537 |  | ATG | T |  | H |
| *cox2* | 1573 | 2397 | 825 | 35 | ATG | TAG |  | H |
| *nad3* | 2405 | 2761 | 357 | 7 | ATG | TAA |  | H |
| tRNA-Ala | 2846 | 2909 | 64 | 84 |  |  | TGC | L |
| tRNA-Ser2 | 2942 | 3007 | 66 | 32 |  |  | TGA | L |
| tRNA-Ser1 | 3021 | 3088 | 68 | 13 |  |  | TCT | L |
| tRNA-Glu | 3104 | 3170 | 67 | 15 |  |  | TTC | L |
| *nad2* | 3196 | 4146 | 951 | 25 | ATT | TAA |  | L |
| tRNA-Met | 4159 | 4222 | 64 | 12 |  |  | CAT | L |
| tRNA-Trp | 4263 | 4327 | 65 | 40 |  |  | TCA | L |
| tRNA-Arg | 4347 | 4411 | 65 | 19 |  |  | TCG | L |
| 12S rRNA | 4412 | 5275 | 864 |  |  |  |  | L |
| tRNA-Lys | 5273 | 5337 | 65 | -3 |  |  | TTT | L |
| tRNA-Thr | 5342 | 5402 | 61 | 4 |  |  | TGT | L |
| tRNA-Tyr | 5415 | 5477 | 63 | 12 |  |  | GTA | L |
| 16S rRNA | 5478 | 6825 | 1348 |  |  |  |  | L |
| tRNA-Leu1 | 6826 | 6897 | 72 |  |  |  | TAG | L |
| tRNA-Asn | 6899 | 6968 | 70 | 1 |  |  | GTT | L |
| tRNA-Pro | 6969 | 7034 | 66 |  |  |  | TGG | L |
| *cytb* | 7043 | 8197 | 1155 | 8 | ATG | TAA |  | L |
| tRNA-Phe | 8207 | 8278 | 72 | 9 |  |  | GAA | L |
| *nad5* | 8315 | 10069 | 1755 | 36 | ATA | TAG |  | H |
| tRNA-His | 10136 | 10210 | 75 | 66 |  |  | GTG | H |
| tRNA-Gln | 10381 | 10454 | 74 | 170 |  |  | TTG | L |
| tRNA-Cys | 10466 | 10535 | 70 | 11 |  |  | GCA | L |
| tRNA-Ile | 10542 | 10611 | 70 | 6 |  |  | GAT | L |
| tRNA-Val | 10644 | 10709 | 66 | 32 |  |  | TAC | L |
| tRNA-Leu2 | 10725 | 10789 | 65 | 15 |  |  | TAA | L |
| *nad1* | 10798 | 11706 | 909 | 8 | ATT | TAA |  | L |
| tRNA-Gly | 11737 | 11802 | 66 | 30 |  |  | TCC | L |
| *nad6* | 11804 | 12301 | 498 | 1 | ATA | TAA |  | L |
| *nad4* | 12357 | 13709 | 1353 | 55 | ATG | TAG |  | H |
| *nad4L* | 13722 | 14033 | 312 | 12 | ATG | TAA |  | H |
| *morf* | 14017 | 14628 | 612 | -17 | ATG | TAG |  | H |
| tRNA-Asp | 14636 | 14713 | 78 | 7 |  |  | GTC | H |
| *atp8* | 14727 | 14909 | 183 | 13 | ATG | TAG |  | H |
| *atp6* | 14962 | 15654 | 693 | 52 | ATG | TAA |  | H |
| *cox3* | 15703 | 16476 | 774 | 48 | ATT | TAA |  | H |
